# Antagonistic and Immunomodulant Effects of Two Probiotic Strains of Lactobacillus on Clinical Strains of *Helicobacter pylori*

**DOI:** 10.31661/gmj.v9i0.1794

**Published:** 2020-10-14

**Authors:** Somayyeh Taghizadeh, Tahereh Falsafi, Rouha Kasra Kermanshahi, Reihaneh Ramezani

**Affiliations:** ^1^Microbiology Department, Faculty of Biological Sciences, Alzahra University, Tehran, Iran; ^2^Department of Biomedical Sciences, Woman Research Center, Alzahra University, Tehran, Iran

**Keywords:** Gastric Epithelial Cell, Helicobacter pylori, Lactobacillus acidophilus, Lactobacillus rhamnosus, Macrophage

## Abstract

**Background:**

The present study aimed to evaluate the in vitro and in situ antagonistic effects of Lactobacillus probiotic strains on clinical strains of *Helicobacter pylori*. Also to investigate their immunomodulation effects on a macrophage cell model.

**Materials and Methods:**

Anti-microbial effects of probiotic lactobacilli against *H. pylori* was assessed using the well and disk diffusion methods. Effects of lactobacilli probiotics strains, as well as their cell-free supernatant on adhesion of *H. pylori* to MKN-45 gastric epithelial cells, were examined in their presence and absence. Immunomodulation effects of probiotic lactobacilli were performed using the U937 macrophage cell model. Incubation of host cells with probiotics and their cell-free supernatants with cultured host cells was performed in different optimized conditions. The supernatant of host cells cultured in their presence and absence was used for cytokines measurement.

**Results:**

Two probiotics,*Lactobacillus acidophilus* ATCC4356, and *Lactobacillus rhamnosus* PTCC1607, could inhibit the growth of clinical *H. pylori* in vitro. They could also inhibit attachment of *H. pylori* to MKN-45 cells. Cell-free supernatant of *L. acidophilus* had a stimulating effect on the production of Interferon-gamma (IFN-γ) by U937 cells.

**Conclusion:**

The present study demonstrates that, *L. acidophilus* ATCC4356 and *L. rhamnosus* PTCC1607 probiotic strains can inhibit the growth of clinical *H. pylori* in vitro. Treatment of U937 with alive *H. pylori* plus cell-free supernatant of *L. acidophilus*, have a significantly higher capacity to stimulate IFN-γ production than *H. pylori* alone. So, the metabolite (s) of this probiotic may have an immunomodulatory effect in immune response versus *H. pylori*.

## Introduction

*H elicobacter pylori*, a flagellated, Gram-negative, microaerophilic, spiral-shaped bacteria isolated from human gastric mucosa was first recognized in 1982. Later it was proposed as the main cause of gastritis and peptic ulcer and a risk factor for gastric malignancy. 25-50% of the population in developed countries and 70-90% of the population in developing countries are infected. The common therapy of *H*. *pylori* infection is‏ the administration of antibiotics plus protons pump inhibitor although, this management is associated with side effects of various drugs, and favors the emergence of antibiotic resistance. Multiple studies have shown a diminution of eradication rates due to resistance to commonly used antibiotics [1-6]. The protective immune response against *H. pylori* is characterized by a strong T-helper 1 (Th1) response. The important step for the generation of adaptive Th1 response would be the interaction of *H. pylori *with surface mucosa which induces the release of proinflammatory cytokine interleukin 8 (IL- 8), leading to the influx of neutrophils, mononuclear cells, and Th1. The neutrophil-activating protein of *H. pylori* stimulates interleukin 12 (IL-12) and interleukin 23 (IL-23) production via activation of Toll-like receptor 2 (TLR2) in antigen-presenting cells (APC) [7]. The investigations have shown that probiotics can be used as a complementary therapy for the management of *H. pylori *infection, since it does not cause side effects [8]. Recently, the utilization of probiotics have proposed as a way to regulate the intestinal bacterial microflora and also suggested as a good approach to prevent cancer, inflammatory diseases through improving the intestinal microflora [9]. Furthermore, probiotics are known for their many beneficial health effects. Clinical experiments have shown useful result for the use of probiotics in different conditions such as inflammatory bowel disease, antibiotic-associated diarrhea, rotavirus infections and atrophy in the infants at risk [10,11]. As; probiotics are useful in the prevention and treatment of certain infections, they may also be considered for *H. pylori* infection. Probiotics play also an important role by regulating the immune system (especially intestinal immune system), host resistance to enteric bacterial pathogens and regulating the environment of intestinal bacteria. The mechanisms involved in these processes are complex and include production of antimicrobial substances, competing with pathogenic microorganisms, preventing pathogenic colonization and invasion, also decreasing the luminal pH value by producing lactic acid. [9,12]. Previous studies on *H. pylori* have shown that probiotics may inhibit its growth independent of lactic acid levels and pH [13]. Multiple investigators have shown that probiotics, predominantly Lactobacillus demonstrate anti- *H. pylori *activity in vitro although it is not clear whether the effects of different probiotics may be similar [12]. Among particular interest related to the probiotic properties of lactobacilli, their ability to modulate the pro-inflammatory, anti-inflammatory immunity and adaptive immune responses, especially the Th1/Th2 and T-regulatory cells (T-reg) balance may be cited. It was also shown that Lactobacillus species have an immuno-enhancing effect on the phagocytic activity of monocytes and polymorphonuclear cells (PMNs) as well as chemokine and cytokine production. These responses are started by the recognition of microbe-associated molecular patterns (MAMPs) through the Toll-like receptors (TLRs) on intestinal epithelial cells (IECs), dendritic cells and macrophage cells [14,15]. Furthermore, multiple studies indicated that some strains of lactobacilli can activate macrophages and induce secretion of *tumor necrosis factor -*alpha (TNF-α), interleukin 1 (IL-1), interleukin 6 (IL-6), (interleukin 12) IL-12, interleukin 18 (IL-18) and IFN-γ. Also, recent studies have shown that, Gram-positive bacteria-induced more Th1-cytokines, e.g. IL-12, IL-18, and IFN-γ, and less interleukin 10 (IL-10) than Th2- cytokines in human monocytes, while Gram-negative bacteria-induced more IL-10 rather than IL-12. The cells that play a vital role in initiating the innate immune response are the macrophages and the dendritic cells [16-18]. Regarding the important roles of probiotics in *H. pylori*-induced infection and their role in immune responses, we aimed to test some strains of Lactobacillus for their in vitro anti-Helicobacter activities and their in situ anti-adhesion effects on gastric epithelial cell model. We also aimed to investigate their immunomodulation effects on the U937 macrophage cell line model.

## Materials and Methods

###  1. Bacterial Strains and Culture Conditions

Nine clinical strains of *H. pylori* obtained from the collection of Alzahra University (Table-1), were grown on Brucella agar (Merck, Germany) supplemented with 5% defibrinated sheep blood and antibiotics under microaerophilic conditions at 37 ˚C for 72 hours, and then identified by biochemical tests and Gram staining. *L.acidophilus* ATCC4356 and *L. rhamnosus* PTCC1607 were obtained from the Organization of Industrial Researches of Iran. * Lactobacillus *spp. were grown on de Man, Rogosa, Sharpe (MRS) agar (Merck, Germany) in an anaerobic chamber under incubation at 37 ˚C.

###  2. In Vitro Antagonist Activity

Lactobacilli strains were cultured in broth media for 48 hours, their supernatant was collected by centrifugation at 6000 rpm (Revolutions per minute) for 30 minutes at 4°C, and filtered through a 0.45 μm (micrometer) filter. Blank MRS broth media was incubated for 48 hours at the same condition, centrifugated at 6000 rpm for 30 minutes at 4°C, and was filtered through a 0.45 μm filter as a control. The anti-microbial activity of probiotic lactobacilli against *H. pylori *strains was assessed using a well and disk diffusion method [19]. In an optimum method, we investigated the effect of three different volumes of probiotic bacterial supernatant (100, 150, 170 microliters) on *H. pylori. *A well containing bacteria-free medium (MRS broth) was used as a negative control. In the disk diffusion method, sterile blank disks were impregnated with the cell-free supernatant of two probiotic bacteria in two volumes of 25 and 50 microliter. To dry them, all disks were placed at 37 °C for 15 minutes. The suspension of *H. pylori* (1 Mc Farland standard) was spread on plates containing Brucella blood agar medium. Impregnated discs were placed on Brucella blood agar with a 2.5 cm distance. Plates were incubated at 37 °C and after 72 hours, the growth inhibition zone of probiotics against *H.pylori* was measured by a ruler. Blank disks were impregnated with bacteria-free MRS broth medium as control.

###  3. In Situ Assay

####  3.1. In Situ Adhesion Assay in Epithelial Host Cells

The gastric epithelial cell line MKN-45 (IBRC C10137) purchased from Iranian Biological Resource Center (IBRC) were cultured in RPMI 1640 (Gibco, UK) containing 2 mM glutamine supplemented with nonessential amino acids and 10% heat-inactivated fetal bovine serum (FBS: Gibco, USA) and antibiotics including penicillin (100 IU/mL), streptomycin (100 mg/mL). The cells maintained at 37°C and 5% CO2 in a wet environment, were seeded into cell culture plates a day before the experiment to form a monolayer. At first, the cell culture medium was replaced with RPMI 1640 with 3% serum without antibiotics. Grown *H. pylori* bacteria from plates were suspended and homogenized in RPMI 1640 to a turbidity of 0.7 optical density (OD) corresponding to a density of 108^8^CFU/ml. Overnight cultures of lactobacillus strains were suspended in RPMI 1640 to an optical density of 1.0. Epithelial cells in 12-well plates (Orange Scientific) were infected with *H. pylori* in the absence or presence of lactobacilli at an MOI (multiplicity of infection) of 100 for each species. A similar infection with *H. pylori* was performed with or without lactobacilli supernatant. After 2 h of incubation, the host cells were washed with sterile phosphate-buffered saline (PBS) three times to remove any unbound bacteria. The host cells were lysed by treatment with deionized sterile water and incubated at 37°C for 30min. The adhesion index was defined as the mean number of adhering bacteria per cell [20].

####  3.2. Immunomodulation Assay in U937 Macrophage Cell Line

U937 cells (ATCC CRL-1593.2), derived from a human histiocytic lymphoma, were obtained from the Iranian Institute of Pasteur. Cells were cultured in RPMI-1640 medium supplemented with 10% heat-inactivated FBS (Gibco, UK), 2 mM glutamine, 10 Units/ml penicillin, and 10 mg/ml streptomycin (Sigma- Aldrich, USA). U937 cells were seeded at a density of 5×105 cells/well in 12-well plates and incubated at 37°C (95% air, 5% CO2 and humidified atmosphere). The viability of the cells (97–98%) was tested by the Trypan blue assay. For all experiments, the U937 cells were differentiated into the macrophages by the addition of phorbol 12-myristate 13-acetate (PMA) (Sigma-Aldrich, USA) at a final concentration of 100 nM for 48 h. Before each experiment, the cells were washed once with sterile PBS buffer to remove all non-adherent cells. One hour before the addition of bacteria, the cell culture media was replaced with RPMI 1640 medium supplemented with 3% FBS (without antibiotics) to allow the cells to adapt to new conditions. Bacteria were added to the host cells at MOIs of 100 cells. As the control, RPMI 1640 medium with 3% (vol/vol) FBS was used. We incubated the cells in five conditions: (i) with supernatant of probiotic bacteria, (ii) with probiotic bacteria, (iii) with alive * H. pylori *bacteria, (iv) with *H. pylori *in combination with probiotics bacteria, (v), and *H. pylori* with cell-free supernatant of probiotic bacteria for 48h. After incubation time, cell-free supernatants of cultured host cells were collected and stored at –80°C until measurements of cytokines.

###  4. Measurements of Cytokines

 IL-4 and IFN-γ levels were measured using enzyme-linked immunosorbent assay (ELISA) by the standard method (R&D systems, USA).

###  5. Statistical Analysis

Each condition was studied in three separate experiments and the values were expressed as mean ± standard deviation. Statistical comparisons between control and treated cell cultures were made by ANOVA followed by paired *t*-.test. A *p*<0.05 was considered significant.

## Results

###  In Vitro Antagonistic Effect of L. acidophilus on H. pylori

**Figure 1:**
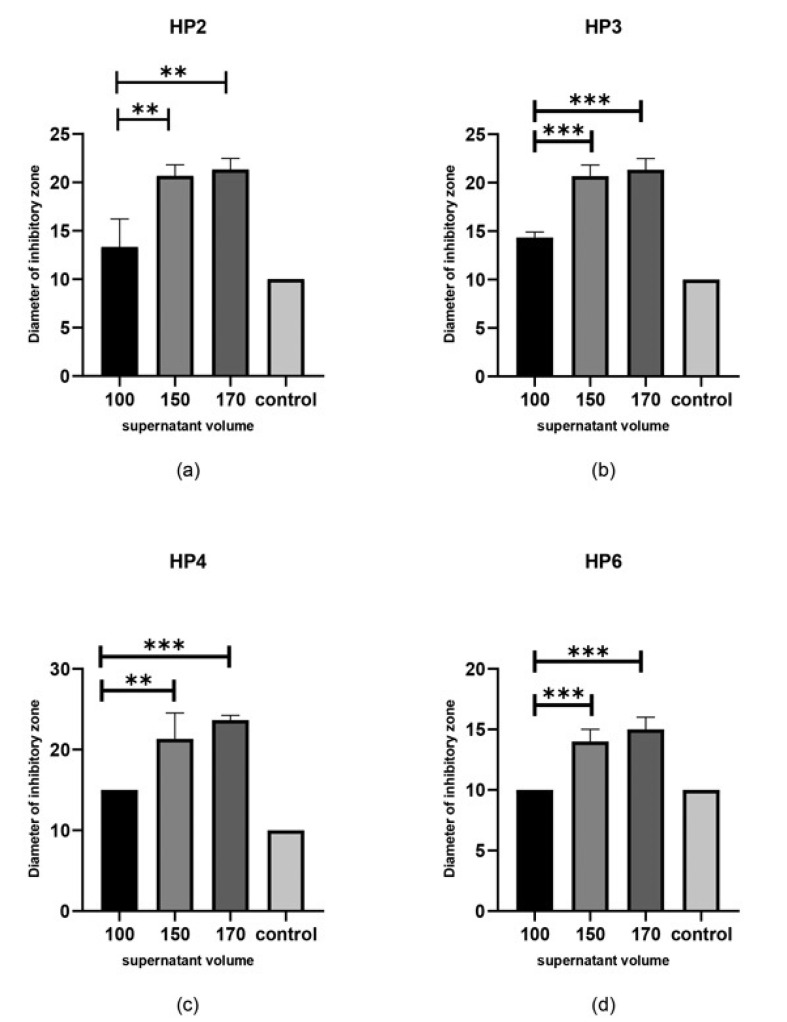
Inhibitory effect of L. acidophilus cell-free supernatant on clinical strain of H. pylori a) HP2, b) HP3, C) HP4, d) HP6 by Well Diffusion Assay on Brucella agar under microaerophilic conditions (5% O2, 10% CO2, and 85% N2) at 37 ˚C for 72 hours. Control: Blank MRS broth. **: P<0.01, ***: P<0.001

- Disk Diffusion: In the disk diffusion method, no inhibitory effects were observed toward all *H. pylori* strains.

- Well diffusion assay with supernatant of * L. acidophilus*: The supernatant of *L. acidophilus *had no significant effect on HP1 and HP5 *H. pylori* strains after 48h. Also, no significant difference was observed between the three different volumes of supernatant. In the case of HP2, HP3, HP4, HP6 H. * pylori *strains, the greatest inhibitory effect was observed in the wells containing 170μl of *L. acidophilus* supernatant (P<0.05), but no differences were found between different volumes of 150 μl and 170 μl (P>0.05). In the cases of HP7, HP8 and HP9 *H. pylori* strains, no inhibitory effects were observed. However, for HP2, HP3, HP4, and HP6, some inhibition was observed (Figure-1).

###  In Vitro Antagonistic Effect of L. rhamnosus on H. pylori

**Figure 2:**
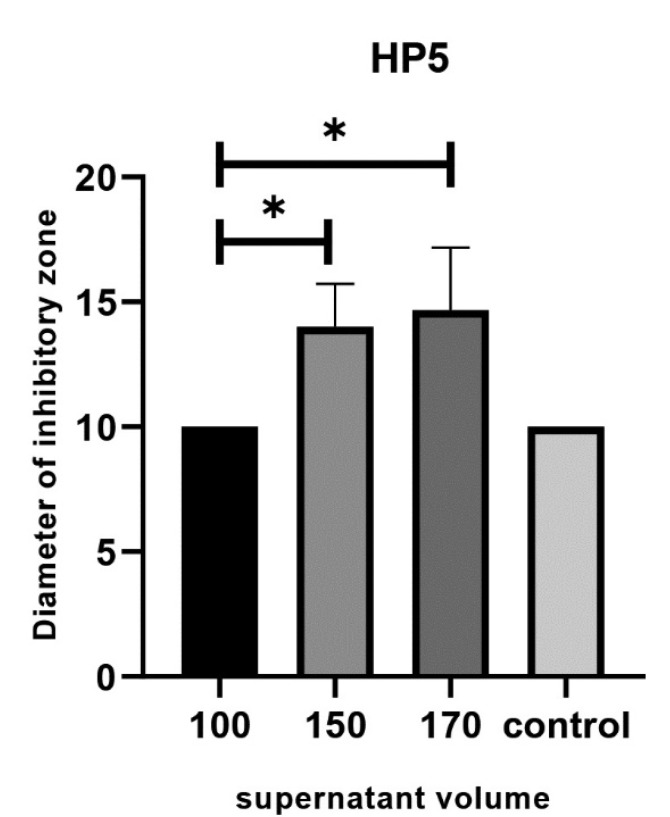
Inhibitory effect of L. rhamnosus cell-free supernatant on clinical strains of H. pylori (HP5) by Well Diffusion Assay on Brucella agar under microaerophilic conditions (5% O2, 10% CO2, and 85% N2) at 37 ˚C for 72 hours. Control: Blank MRS broth. *: P<0.05

For HP1, HP2, HP3, HP4, HP6 strains comparison of various amounts (volumes) of *L*. *rhamnosus* supernatant showed no significant differences after 48h incubation. For HP5 strain, the supernatant of *L. rhamnosus* showed the most inhibitory effect using 170μl of *L. rhamnosus* supernatant (P<0.05, Figure-2), but no significant differences were found between the amounts of 150 μl and 170 μl (P>0.05). For HP7, HP8 and HP9 strains, no inhibitory effects were observed.

###  Adhesion Assays with MKN45 Gastric Epithelial Cell Line

The actual adhesion index for *H. pylori* strains and inhibitory effect of Lactobacillus strains against adhesion of *H. pylor*i various strains are shown in Table-2. *L. rhamnosus* showed no anti-adhesion effect in the case of HP4, HP1, HP2, while *L. acidophilus* showed the highest anti-adhesion effect in the case of HP7, HP8, and HP9. Comparison of adhesion index of * H. pylori *strains (Table-2: column 4) in absence of probiotics with those of in their presence (Table-2: columns 2-3) showed the highest anti-adhesion effect for HP6 by *L. acidophilus.*

###  Immunomodulation Assay Using U937 Macrophage Cell Line

**Figure 3:**
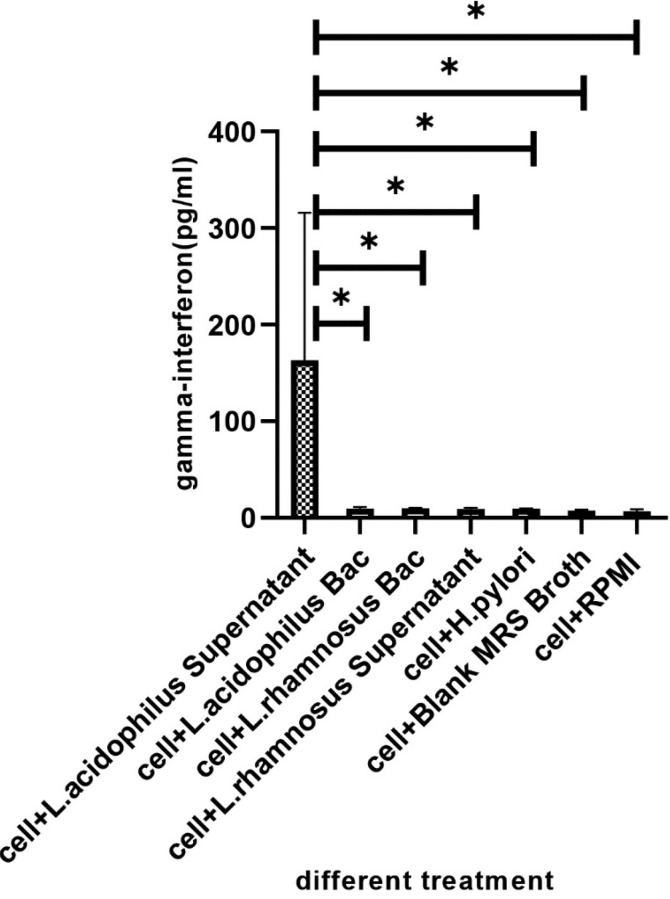
Comparison of various conditions with control for IFN-γ secretion by macrophage cell line U937. The result was expressed as a mean of three independent experiments± SD. Statistically significant differences with the negative control are indicated as: *: P<0.05. Bac: bacteria.

**Figure 4:**
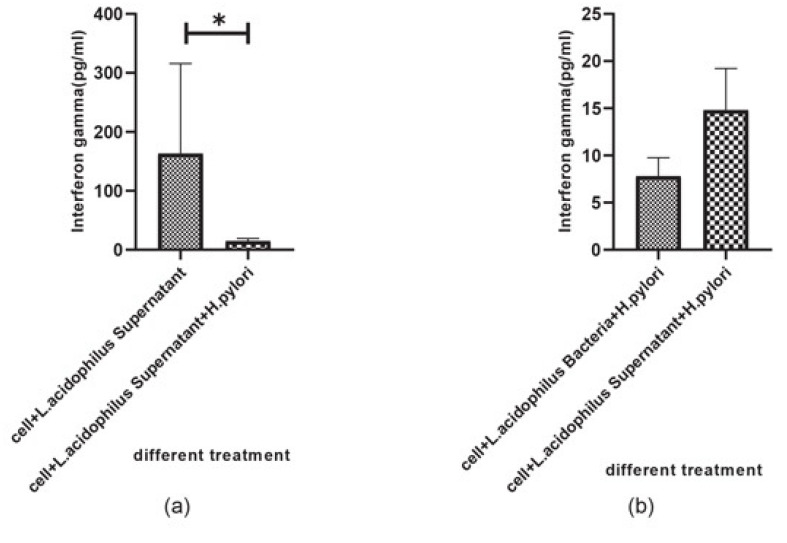
Comparison of IFN-γ secretion by U937 cell treated with L. acidophilus bacteria and the cells treated with cell-free supernatant. The result was expressed as the mean of three independent experiments± SD. Statistically significant differences with the negative control are indicated as *: P<0.05. (Bac: bacteria)

**Figure 5:**
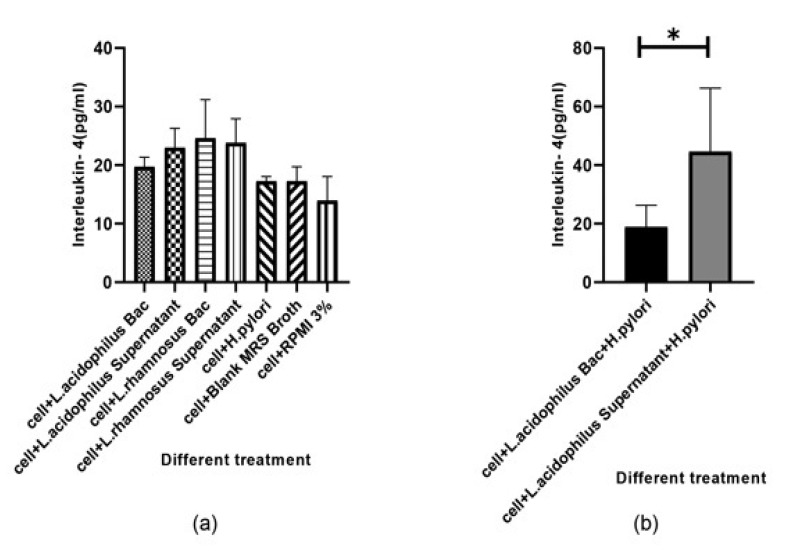
Comparison of various conditions with control for IL-4 secretion by macrophage cell line U937. The result was expressed as a mean of three independent experiments± SD. Statistically significant differences with the negative control are indicated as: *: P<0.05. Bac: bacteria.

The amounts of IFN-γ and IL-4 produced by U937 cells was measured by ELISA in the cell-free supernatants of U937 cells after 48h incubation. The monolayers of U937 cells were incubated in five conditions: (i) in presence of alive * H. pylori* alone, (ii) in presence of *L. acidophilus* ATCC4356 and *L. rhamnosus* ATCC alone, (iii and iv), with supernatant of probiotic bacteria, and (v) with supernatant of probiotic bacteria plus alive *H. pylori*. Our results showed that supernatant of * L. acidophilus* ATCC4356 stimulated markedly the production of IFN-γ (Figure-3). Also comparing the case of *L. acidophilus* supernatant with that of *L. acidophilus* plus live *H. pylori* showed a significant increase in IFN-γ production (P<0.05). Furthermore, the supernatant of *L. acidophilus* demonstrated a higher ability to enhance IFN-γ production by the U937 cell line in comparison with that of *L. rhamnosus* (Figure-3). In other words, *L. acidophilus* supernatant demonstrated a higher ability to induce IFN-γ production than supernatant plus alive *H. pylori* (Figure-4). The central finding is that IFN-γ production in U937cell infected with *H. pylori* plus supernatant. Analysis of variance showed no significant effect on the production of IL-4 by U937 cell type in similar treatments (P>0.05, Figure-5). But in the case of *H. pylori* plus *L. acidophilus* supernatant also * H. pylori* plus *L. acidophilus *bacteria, our results showed important change.

## Discussion

Numerous in vitro studies have shown that probiotic Lactobacilli have anti-Helicobacter activity. Among them, Bhatia *et al* [21] showed that Lactobacillus strains have an antagonistic in vitro effect on the clinical *H. pylori*. Michetti, *et al* [22] have reported the inhibitory in vivo effect of Lactobacillus strains on *H. pylori* infection. O’Connor *et al* have also shown that some probiotics, such as Lactobacilli and Bifidobacteria have anti– *H. pylori* effects in vitro and may help reduce antibiotic-related side effects [23]. The present study demonstrates that two standard probiotic strains can inhibit the growth of clinical *H. pylori* in vitro which could be due to the production of organic acid. Using standard strains of *L. acidophilus* and *L. rhamnosus*, we also tested theirs in vitro inhibitory effects on the growth of *H. pylori* and we observed an inhibition toward the growth of * H. pylori*. We also tested the anti-adhesion effects of the probiotics on adhesion of *H. pylori *to the MKN-45 epithelial cell model. A comparison of the adhesin index of *H. pylori* strains to MKN-45 with those of *H. pylori *in the presence of probiotics showed that both *L. acidophilus* and *L. rhamnosus* have anti-adhesion effect on *H. pylori *strains (Table-2). Previous results have been obtained on the direct inhibitory effect of Lactobacillus strains to reduce the capacity of *H. pylori* adhesion to the host cells [24]. An in vitro study by Kabir and colleagues has shown that *Lactobacillus salivarius* WB 1004 can inhibit the attachment of *H. pylori* to gastric epithelial cells (in both murine and human) [25]. However, we observed that inhibition of various *H. pylori* strains by lactobacilli was not similar. This may be since clinical strains of *H. pylori *isolated from the patients with the various pathology of *H. pylori* infection were not similar genetically. This inhibition may be ensured via binding of probiotics to the same site and preventing attachment and colonization of the *H. pylori*. It was proposed that cellular immune responses have a key role in protective immunity against *H. pylori* infection. Multiple investigations have shown that the Th1 response promoting key cytokines production such as T-cells from *H. pylori-*infected persons expressed a higher proportion of IFN-γ than gastric T cells from uninfected persons [26-30]. Also, contact between *H. pylori *and the macrophages stimulates macrophages to the production of several cytokines [31-33]. However, * H. pylori *impair the killing activity of macrophages which may be through the mechanisms such as expression of catalase since, in comparison with a wild-type catalase-positive *H. pylori *strain, catalase-deficient strains have been more susceptible to macrophage killing [34]. Another mechanism may be through blocking of nitric oxide production which is mediated by *H. pylori *arginase, which competes with nitric oxide synthase for arginine [35]. In addition to resistance to be killed by macrophages, *H. pylori *can induce macrophage apoptosis by activation of arginase II pathway [36-38]. We investigated the immunomodulation effect of the mentioned probiotic strains in the U937 macrophage cell model. We did not observe a macrophage killing effect toward *H. pylori* strains. Furthermore, our results showed that the supernatant of *L. acidophilus* ATCC4356 strain stimulated IFN-γ production which was more than the case of *L. rhamnosus* PTCC1607 (Figure-3). We also observed that, the cell-free supernatant of *L. acidophilus* had a stimulating effect on the production of IFN-γ by U937 cells which were higher than this lactobacillus alone. It may be concluded that production of IFN-γ is due to the presence of a (the) metabolite (s) in the supernatant of *L. acidophilus*. As, IFN-γ plays a key role in the induction of Th1 immune pathway, it may be concluded that it improves the immune response against *H. pylori* infection. Multiple investigators have studied the effect of probiotics on the immune system and have observed that some strains of LAB can induce the secretion of cytokines such as IL-12 which induces IFN-γ production in favor of T-helper 1 pathway [39,40]. Maassen *et al*. have reported that treatment of mice with L. reuteri and L. brevi causes an increase in expression of the pro-inflammatory/Th1 cytokines including IL-2, TNF-α and IL-1b [41]. But contradictory results were obtained about the immunomodulant roles of probiotics in increasing some cytokines including IL-10, IFN-γ, IL-1b, IL-6 and TNF-α production, as well as IgA production [42-46]. Recently, it was found that *Lactobacillus *sp. strains have an antagonistic effect on *H. pylori *[47-49]. It was demonstrated that *L. acidophilus *induces the production of IFN-γ by murine peritoneal macrophages [50,51]. Also, *L. bulgaricus *and *Streptococcus thermophiles* could induce the production of IL-1β, TNFα and IFNγ by peripheral blood mononuclear cells in humans, the cytokines which were also induced by *L. casei*, *L. acidophilus*, *Bifidobacterium *spp., and *L. helveticus* [52]. The conclusion that may be highlighted by our observation, is that the treatment of U937 with alive *H. pylori* plus cell-free supernatant of *L. acidophilus*, have a significantly higher capacity to stimulate IFN-γ production than *H. pylori *alone (Figure-3). So, the metabolite (s) of this probiotic may have an immunomodulatory effect in the immune response. It should be noted that during the treatment of U937 cells with *H. pylori* plus supernatant of *L. acidophilus*, the IFN-γ production rate showed small change compared to the control samples. (Figure-4). It may be hypothesized that the acid produced by *L. acidophilus* might reduce the pH of culture medium and *H. pylori* produce urease to cope with the acidic condition, thereby neutralizing the environment. Studies by Sgouras *et al*. have shown that Neutralization of local acidity in favor of H. pylori survival exerted by *H. pylori* urease, that way urease catalyzes the conversion of urea to carbon dioxide and ammonia; ammonia, in turn, forms ammonium hydroxide, which neutralizes the local acidity while some studies reported the *Lactobacillus casei* can inhibit H. pylori urease. [53,54]. Our data clearly showed that the cell-free supernatant of *L. acidophilus* stimulates the U937 cell line to produce more IFN-γ than *L. acidophilus* alone. It is concluded that organic acid production by lactic acid bacteria can have an important role in anti- *H. pylori *activity [54-56]. Although the inhibitory effects observed with *Lactobacillus acidophilus* might be a result of organic acids production but other metabolites may also be involved that include small peptides, bacteriocins and non-proteinaceous molecules that can show direct bactericidal effect as the case of a 45 kDa S-layer protein on the surface of *L*. * acidophilus* NCFM strain can stimulate dendritic cell to cytokine production [57-58]. We observed that U937 macrophage cells treated with *L. acidophilus* supernatant plus alive *H. pylori*, demonstrated a significant difference in IL-4 production in comparison with those treated with live *L. acidophilus* and *H. pylori*, so it can be interpreted that cell suspension with *L. acidophilus* supernatant alone stimulates the production of IFN-γ. However, this soup plus live *H. pylori* did show no increase in IFN-γ production, due perhaps to the urease activity of *H*. *pylori*, whereas this combination stimulated IL-4 production in U937 macrophage cell. Therefore, it can be concluded that another factor (metabolite other than presumed acid) in *L. acidophilus* can stimulate the production of IL-4. This stimulation may promote the humoral immune response, which is recommended for further investigation. Also, IL-4 production was higher in the case of *L. rhamnosus* and its supernatant than other treatments although it was not significant. So, it may be proposed that *L. rhamnosus* can stimulate humoral Immune response rather than the cellular one.

## Conclusion

The present study demonstrates that *L. acidophilus* and * L. rhamnosus* can inhibit the growth of clinical *H. pylori* in vitro and treatment with alive *H. pylori* plus cell-free supernatant of *L. acidophilus,* have a significantly higher capacity to stimulate IFN-γ production by U937 than *H. pylori *alone. So, the metabolite (s) of this probiotic may have an immunomodulatory effect in the immune response.

## Acknowledgment

 This study was supported by the Department of Microbiology, College of Biological Sciences, Alzahra University, Tehran, Iran.

## Conflict of Interest

 The authors have no conflicts of interest to disclose

**Table 1:** Strains Used in this Study

**Isolate**	**Histopathological status**	**Gender**	**Age (year)**
**HP1**	Sever active chronic gastritis	Female	4
**HP2**	Sever chronic gastritis	Male	9
**HP3**	Moderate chronic gastritis	Male	9
**HP4**	Moderate chronic gastritis	Female	11
**HP5**	Severe chronic gastritis	NA	NA
**HP6**	Moderate chronic gastritis	NA	NA
**HP7**	Moderate chronic gastritis	NA	NA
**HP8**	Moderate chronic gastritis	Female	13
**HP9**	Moderate chronic gastritis	NA	NA

**NA:** Not available

**Table 2:** Inhibitory Effect of Lactobacillus Strains against Adhesion of H. pylori Strains to MKN-45 Gastric Epithelial Cell Line

**H. pylori strains**	**H. pylori + L. rhamnosus Adhesion index**	**H. pylori + L. acidophilus Adhesion index**	**Adhesion index of H. pylori strains**
**HP1**	0.75	0.55	0.01
**HP2**	0.25	0.005	1.57
**HP3**	0.25	0.58	0.64
**HP4**	0.77	0.42	3.5
**HP5**	1.15	0.22	0.22
**HP6**	1.03	0.1	1
**HP7**	0.37	0.01	0.02
**HP8**	0.56	0.02	0.01
**HP9**	0.07	0	0.009
